# Determination of Pesticide Residues in Cannabis Smoke

**DOI:** 10.1155/2013/378168

**Published:** 2013-05-12

**Authors:** Nicholas Sullivan, Sytze Elzinga, Jeffrey C. Raber

**Affiliations:** The Werc Shop, Inc., Pasadena, CA 91107, USA

## Abstract

The present study was conducted in order to quantify to what extent cannabis consumers may be exposed to pesticide and other chemical residues through inhaled mainstream cannabis smoke. Three different smoking devices were evaluated in order to provide a generalized data set representative of pesticide exposures possible for medical cannabis users. Three different pesticides, bifenthrin, diazinon, and permethrin, along with the plant growth regulator paclobutrazol, which are readily available to cultivators in commercial products, were investigated in the experiment. Smoke generated from the smoking devices was condensed in tandem chilled gas traps and analyzed with gas chromatography-mass spectrometry (GC-MS). Recoveries of residues were as high as 69.5% depending on the device used and the component investigated, suggesting that the potential of pesticide and chemical residue exposures to cannabis users is substantial and may pose a significant toxicological threat in the absence of adequate regulatory frameworks.

## 1. Introduction

Cannabis *sativa* L. has been widely utilized by humans for thousands of years for the relief of a wide range of physiological ailments. In the United States, there are currently 18 different states and the District of Columbia that legally allow for the medical use of cannabis, and most recently the states of Colorado and Washington have legalized the use of cannabis by adults for recreational purposes. State lawmakers and regulatory departments are now being tasked to best enact appropriate laws, rules, and regulations on the use of cannabis for both medicinal and recreational purposes. While medicinal use of cannabis in a smoked form may be widely debated as an effective delivery form, rapidity of effect and ease of titration of dose lend it to be extensively used by many patients as their preferred delivery method today. Undoubtedly, recreational use will see considerable consumption via smoking of dried cannabis flowers. In an effort to help aid patients, lawmakers, regulators, and the general public understand the potential harms of contaminated cannabis we sought to determine to what extent pesticide residues may transfer into the mainstream smoke, produced from cannabis, when inhaled through various smoking devices currently being used by medical cannabis patients. Mainstream smoke consists of the smoke inhaled from a smoking device directly while sidestream smoke refers to smoke that otherwise escapes the device and is not directly inhaled.

The ubiquitous use of pesticides in agriculture has earned itself a long history in the United States from the outset of the Insecticide Act passed in 1910 to the now heavily engaged US Environmental Protection Agency (US EPA), Federal Department of Agriculture (FDA), and United States Department of Agriculture (USDA) along with individual state regulators [1]. According to a report issued by the US General Accounting Office (GAO) in 2003, the use of pesticides on tobacco crops was limited to 37 pesticides, which included various organochlorides, organophosphates, and other classes of pesticides. Allowable pesticides and residue levels on food crops are determined by the US EPA, while the testing and monitoring of the presence and levels of residues are conducted by the FDA and USDA. However, since tobacco is not a food crop, the US EPA has not set tolerances on the residue levels on tobacco crops. Consequently, tobacco is only monitored for compliance with US EPA approved pesticides while the residue levels are not federally regulated [2].

To date, there are no approved pesticides or application limits established for use on cannabis crops by the US EPA; therefore, all pesticide use on this crop is currently illegal [3]. The use of pesticides and plant growth regulators in medicinal cannabis cultivation has been found to be quite prevalent by both testing laboratories and authority laboratories alike. Many commercially available pesticide containing products or nutrient systems, some only approved for use on ornamental crops, are widely available from a variety of sources including hardware stores, specialty indoor hydroponic shops, and various, sometimes unscrupulous, online vendors. While 18 states allow cannabis for medicinal use, the majority of the current medical cannabis supply lacks regulations and enforcement related to the quality and safety of the plant material for consumption. Laboratories operating within California have reported that cannabis samples contaminated with residual pesticides are frequently encountered. In 2009 the Los Angeles City Attorney's office covertly acquired and then tested three medical cannabis samples available to patients through dispensaries and found that in two of the samples exceedingly high levels of bifenthrin were found. In one sample, 1600 times the legal digestible amount was measured, and in the other, 85 times the legal limit was measured, although the exact quantities were not stated [4].

Many medical cannabis products are currently cultivated, processed, and prepared by private entities that are not regulated by external agencies. The lack of quality control results in patients potentially being exposed to cannabis contaminated with toxic levels of pesticides. Although not yet directly quantified, additional health complications in patients may become a contingency of pesticide exposure and may also interfere with long-term cannabis use studies. Regardless, pesticide toxicity is well documented [5] and more importantly can pose substantial threats to immunocompromised patients or patients with other conditions, such as diseases of the liver, that may intensify the toxicological effects of pesticide exposure [6]. Additionally, during heating pyrolysis products from the plant material form a highly complex mixture of products, many of which may interact with the pesticides or pyrolysis products of the pesticides forming more toxic materials, or highly toxic pyrolysis products may form from the pesticide residues alone [7]. As stated in the review by US General Accounting Office (GAO) in 2003, exposure to organophosphate pesticides through inhalation causes the most rapid appearance of toxic symptoms, and the primary cause of death from organophosphate pesticides is respiratory failure [2]. Considering these issues, evaluation of the exposure from contaminated cannabis needs to be urgently addressed so that new regulations can be properly guided.

A previous pesticide study conducted with filtered tobacco cigarettes had positively identified the recovery of pesticides in the mainstream smoke to range from 2 to 16% [8]. Additionally, the distributions of volatilized pesticides and pyrolysis products in tobacco cigarette mainstream smoke and sidestream smoke were found to differ [7]. The mainstream smoke pesticide residues consist primarily of unpyrolized pesticides carried over by distillation characteristics related to steam volatility, while in the sidestream smoke, a larger portion of pyrolysis products are found [7]. In the same study, it was determined that about one half of ^14^C-labeled pesticides were retained in a cotton cigarette filter in a nonselective manner [7]. For the most part, since cigarette filters absorb a significant portion of the volatilized residues and a substantial toxicological threat is already associated with smoking tobacco, little concern for pesticide exposure to tobacco smokers has been considered [2, 7]. Cannabis smoking devices often do not include filtration processes and because of this the potential quantities of pesticide residues that may be consumed increases dramatically when compared with tobacco smoking. In the present study, we chose to evaluate both filtered and nonfiltered smoking devices to better understand this effect with cannabis and commonly employed medical cannabis consumption methods. While it is known that combustion of plant material causes the formation of carcinogens, there has been no direct correlation in the formation of lung cancers to the inhalation of combusted cannabis [8]. The presence of pesticide residues is therefore critical to be monitored, and furthermore, those individuals seeking to use cannabis for medicinal purposes may also be more physiologically susceptible to negative impacts caused by the presence of these residues.

To prevent overtreatment of tobacco with pesticides, certain application limits on crop treatment have been imposed to minimize exposure to tobacco smokers, but these are not fully federally regulated [2, 9, 10]. Industrial and other laboratories have attempted to quantify the levels at which pesticide residues transfer into the smoke stream in order to validate what quantities of pesticides may safely be applied to crops, and these values have been used to help moderate the levels of pesticide exposure of the public [5, 11]. Considering that there currently exists a significant lack of analogous regulations set in place for the medical cannabis supply, it is important that the potential for pesticide exposure is evaluated under conditions commonly employed by the medicinal user. In order to determine the existence of pesticide and chemical residues in the cannabis smoke stream, a number of pesticides and a plant growth regulator which are readily available to cannabis cultivators and have been measured in high frequency in various medical cannabis products (unpublished data, The Werc Shop, Inc., 4) were selected for the study. Three different smoking devices, chosen to provide a broad overview, were used in the study; a small glass pipe, a water pipe, and an identical water pipe outfitted with activated carbon filters and cotton filters.

## 2. Methods

### 2.1. Chemicals

Acetonitrile, methanol, and water of analytical grade as well as washing acetone and methanol of laboratory grade were purchased from Sigma Aldrich, St. Louis, MO, USA. Bifenthrin and diazinon were purchased from Chem Service, West Chester, PA, USA. Paclobutrazol and permethrin were purchased from Sigma Aldrich, St. Louis, MO, USA. Virgin coconut carbon and cotton were obtained from Scientific Inhalations, Grass Valley, CA, USA.

### 2.2. Smoking Devices

The water pipe was manufactured by Scientific Inhalations, Inc. and is named the McFinn Triple Filtered Water Pipe having a vapor flow path consisting of first a 2.5 cm cup for placement of the flower material, followed by a 2.5 cm connector, flowing in to a 10 cm filter, down further into a 15 cm water chamber having a 3.1 cm inner diameter and a water fill line 3.8 cm from the base. The water chamber also has a second 12.5 cm filter chamber connected at a 45° angle through a 5 cm fitting that is located 12.5 cm above the base of the water chamber, and the second arm then further connects to a mouth-piece. A special mouth-piece was custom made by Scientific Inhalations to allow for easy connection to the gas-wash bottle apparatus. The glass pipe was custom made by Scientific Inhalations to be 10.5 cm long with a 3.1 cm chamber diameter and 1.1 cm inner diameter that included a special mouth-piece configuration for easy adaption to the gas-wash bottle apparatus.

### 2.3. Method for Identification and Quantification of Pesticide Residues by GC-MS

Analysis was conducted with a GCMS-QP2010 PLUS (Shimadzu, Japan) gas chromatograph-mass spectrometer. Separations were performed using a Shimadzu SHRXI-5MS 30 meter, 0.25 mm i.d., and 0.25 um film thickness column. Gas chromatography parameters were as follows: injector temperature 250.0°C, splitless injection mode, column oven temp. 50.0°C held for one minute, followed by an increase to 125°C by 25°C/min, and finally increased to 300°C for 15 minutes by 10°C/min. The column flow was set to 1.69 mL/min 99.999% Helium. MS scan was carried out in selected ion monitoring (SIM) mode with two reference ions for each pesticide to avoid false positives from the complex matrixes. Pesticide calibration curves were prepared in matched matrixes, which were prepared from unspiked plant material using the same smoking procedure used for all the experiments as described in Section 2.6.

### 2.4. Preparation of Pesticide Spiked Plant Material

Plant material was prepared by first placing approximately 8 grams of homogenized cannabis flower material into a 250 mL round bottom flask and vortexed at 1200 rpm until the small non-leafy material fell to the bottom. This material was then separated and sifted over a rough screen to further remove small non-leafy material. This process was repeated five times until the plant material was sufficiently cleared of fine material that might otherwise incur poor homogeneity of pesticide distribution in the bulk of the material.

To the sifted plant material, a concentrated solution of pesticide mixture in methanol, prepared to contain 0.730 mg/mL bifenthrin, 7.41 mg/mL diazinon, 4.37 mg/mL paclobutrazol, and 6.18 mg/mL permethrin, was then added incrementally to the plant material. These concentrations were selected to allow for full quantification of residues captured in the gas wash bottle solutions. A total of 8.30 mL of the pesticide mixture solution was added to 7.4860 g of the material incrementally. Each increment was carried out by adding 1 mL of the solution drop-wise into a 250 mL round bottom flask containing the plant material that was then vortexed at 1300 rpm over a 2 minute period. After each mL was added, the flask was then placed on a rotary evaporator and rotated at 50 rpm for 3 minutes while under vacuum. This was repeated until all 8.30 mL were added and then evaporated. The flask was then covered in a dark encasing and stored at −20°C until further used. From the spiked plant material, duplicate samples were prepared and evaluated for homogeneity of the pesticide distribution. The measured values were averaged and this value was used for the recovery calculations in the smoke condensate.

### 2.5. Apparatus and Method for Condensation and Recovery of Pesticide Residues in Smoke Stream

The smoke stream was collected by being directed through two gas washing bottles which were placed in tandem cold methanol traps both held at −48°C. The gas wash bottles were filled with 100 mL of analytical grade methanol each. The gas wash bottles were then connected with a 6 inch tube in tandem to a vacuum pump intermediated by a gas flow regulator. The end of the system was then fixed to the smoking devices via a frosted glass fitting or direct connection via tygon tubing. A vacuum was applied to the system using a diaphragm vacuum pump (MD 4C, Vacuubrand, Essex, CT, USA) in order to pull smoke from the smoking device and through both of the gas wash bottles.

In order to ensure that the draw rate and vacuum pressure were constant throughout all experiments, a simple device was arranged to monitor the vacuum settings. A long glass column was placed upright in a water vessel filled with a constant volume of water. To the top end of the glass column, a tubing fitting was fixed and vacuum tubing connected. To the tubing, a valve at a constant setting was opened slightly to allow air to enter and prevent the water from being pulled into the vacuum. After having twelve different current medical cannabis patients inhale through the end of a tube attached to the valve while instructed to emulate the draw strength they typically use for these smoking devices, it was determined that the draw rate of an average smoking device user was approximately 1.2 L/min. This draw rate was then used for all of the experiments by ensuring that the vacuum was set to draw at a rate that yielded height in the water column corresponding to 1.2 L/min. This process was performed before, during, and after each experiment to ensure the simulated inhalation flow rate was as consistent as possible.

### 2.6. Smoking Procedure

The smoking procedure was carried out by passing the flame of a disposable lighter over the plant material for three seconds at 15-second intervals while the vacuum was applied at 1.2 L/min. For each experiment, approximately 0.45 g of spiked cannabis was used. Aliquots from the gas wash bottles were taken after being shaken and agitated to capture any condensate on the walls and stems of the wash bottles and measured with GC-MS. Samples were then stored at −20°C in the absence of light. All glassware, tubing, and smoking devices were then washed thoroughly with methanol and acetone between experiments. In the case of the water pipe, water was used in the water chamber as per manufacturer's specifications, and when applicable, 7.5 g of virgin coconut carbon was used in the carbon filter cartridge, while 0.7 g of cotton was used in the cotton filter cartridge. After each experiment using the filtered device, the cotton and carbon were extracted with 15 mL of analytical grade methanol and measured by GC-MS. Experiments were carried out in triplicate for each device.

### 2.7. Preparation of Calibration Curves

Three sets of calibration curves were prepared, each in different matrixes that consisted of smoked plant material solutions in order to account for possible ion suppression from the matrixes. All matrixes and plant material samples were ensured to be free of the pesticides of interest before use and further analysis. For the preparation of the raw plant material matrix, approximately 4 g of unspiked cannabis plant material from the same source as that which was spiked was extracted with 100 mL of analytical grade methanol and stirred with a stir bar for 20 minutes, followed by filtration through a Buchner funnel. Smoke condensate matrixes from the glass pipe and the water pipe were prepared by running the experiment with each device as described in Section 2.6 and storing the solutions in a dark container at −20°C before analysis. Each of these matrix solutions was then used to dilute the stock solutions of pesticides for generating calibration curves in each matrix.

## 3. Results

**Figure 1:**
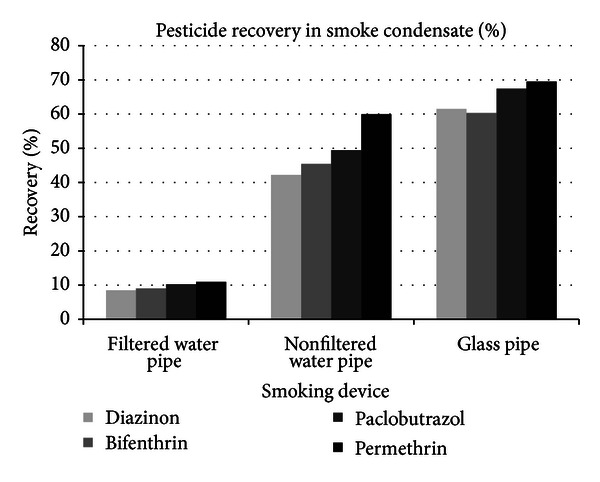
Percent recovery of pesticides from the smoke stream from each device.

The calibration solutions of chemical residues were prepared in the three separate matrixes and the calibration curves generated are tabulated in Table 1.  Table 2 presents the chemical residue content of the spiked plant material. Chemical residues recovered from the smoking devices are tabulated in Table 3, as well as the percent recovery with respect to the spiked plant material. It should be noted that 97% of the recovered residue in the gas wash bottles was found in the first wash bottle, representing excellent recovery capabilities. In all three experiments, the recovery of chemical residues from the activated charcoal was below the lowest calibration level and is therefore not reported.  Figure 1 illustrates the comparative recovery of chemical residues from each of the smoking devices.

## 4. Discussion

The relative amounts of pesticide residues present in other smoked plant material, most notably tobacco, have been studied to determine the amount present in raw plant material, as well as the levels of transfer into the smoke stream. These results have been used to help guide regulations on pesticide application on tobacco crops and reduce the potentials of pesticide toxicity in consumers [9, 12, 13]. As medical cannabis patients already possess negative health complications, exposure to pesticides may create additional health complications and interfere with other health care approaches. In addition, the awareness of proper and safe pesticide use and application is very important to any crop that will be consumed, especially one that will be inhaled. Understanding to what extent chemical residues may be consumed by the user of the final product is important, but also improper applications of pesticides on cannabis crops may lead to other contingencies such as applicator exposure and environmental contamination. To bring attention to the importance of pesticide awareness and to further the regulatory efforts for both the medical cannabis and impending recreational cannabis supplies, the present study demonstrates quantitatively the potential for pesticides to be transferred into the smoke stream under the conditions often encountered by cannabis users. While the variance between triplicate samples was notable, when considering the vast number of variables including heating conditions, and other inherent variations, the overall variation was fairly minimal.

From the data presented here, the recoveries of pesticide residues in the smoke stream are very significant in relation to the potential of exposure by the end consumer. A previous study with filtered tobacco cigarettes published by Cai et al. [9] noted that the range of pesticide recovery from the smoke stream was 2 to 16%. The range of pesticide residue recovery in that study was comparable to the water pipe with filters (0.08–10.9%) used in the present study, but without filters the recovery from the present study was much higher as evident in Table 3 and Figure 1. This suggests that the cotton filters in a cigarette or water pipe are critical in capturing and reducing pesticide residues in the mainstream smoke. Also, extractions of the cotton filters (Table 3) contained a significant portion of the pesticides passed through the device. The carbon filter retained an insignificant amount of pesticides, but this may have been due to heating and desorption of retained compounds during each use as this portion is closest to the plant material combustion point. Between the glass pipe and the water pipe with no filters, the relative pesticide recovery was greater when the glass pipe was used. This difference may be attributed to the comparable levels of surface area for the residues to accumulate inside the device by condensation, as well as factors such as total path length, smoke stream total flow rate velocity, and the absolute temperatures achieved in situ. Additionally, the water pipe contained room temperature water that aids in cooling the smoke stream before exiting the device. Comparative recoveries between individual pesticides (Figure 1) show significant differences in the recovery of each pesticide. These differences may be attributed to the variations in stability of each compound, volatilization characteristics, and to what extent degradation occurs during heating and combustion of the plant material surface.

It should be noted that different levels of pesticides present on different varietals of cannabis flowers present different matrixes that may impact the amount of pesticides potentially being inhaled. Different user behaviors including depth of breath, length of inhalation hold time, and choice of heating method may also impact overall individual exposure amounts. In our lab we use validated methods to detect pesticides above EPA-based acceptable daily intake levels for a 40 Kg individual consuming 10 g of flower material per day. While these limits represent residues on plant material at levels lower than the levels utilized in this study, a number of samples seen have failed considerably further supporting previous findings by local authorities [4]. Additional efforts are ongoing to quantify the amount of pesticides being detected in contaminated medical cannabis products.

## 5. Conclusion

The present study clearly demonstrates that chemical residues present on cannabis will directly transfer into the mainstream smoke and ultimately the end user. Recoveries occurred in the highest quantity with the hand-held glass pipe, ranging between 60.3% and 69.5%. Recovery from the unfiltered water pipe ranged between 42.2% and 59.9%, and recovery from the filtered water pipe ranged between 0.08% and 10.9%. As mentioned previously, the effects of filtration have a significant impact on the total residues consumed. While there are differences between the devices, in general the portion of pesticide recovery is alarmingly high and is a serious concern. Although pesticides are designed to degrade fairly quickly in the environment [14], it is evident from this study that some are highly resistant to pyrolysis and volatilize easily into the smoke stream in agreement with previous studies noting the distillation behavior of pesticides in mainstream smoke [7]. Considering these results, high pesticide exposure through cannabis smoking is a significant possibility, which may lead to further health complications in cannabis consumers. This revelation certainly confounds previous metastudies seeking to determine the possible negative consequences associated with long-term cannabis use, as our experience with a breadth of samples indicates a significant possibility that the negative consequences reported in these studies could have been the result from various chemical residue exposures resulting from the use of unregulated product supply chains. As more states legislate and regulate cannabis products, a strong regulatory approach will help to reduce the potential public health and safety consequences from pesticide exposure. While it is fortunate that chemical residue recovery may be minimized with smoke filtering, this only serves to improve consumer safety today with no adequate regulations, as there is no better way to avoid pesticide and other chemical residue consumption than to assure it is not present on the product in the first place. Active sampling and analytical monitoring of the cannabis supply, along with collaborative efforts between current patients and state regulatory authorities, are needed to help further guide the development and implementation of proper application methods and testing standards that will avoid environmental contamination and consumer threats to public health and safety.

## Figures and Tables

**Table 1:** Calibration curves and goodness of fit values.

Residue	Range (*μ*g/mL)	Raw plant material matrix	Glass pipe smoke matrix	Water pipe smoke matrix
Diazinon	0.737–36.9	0.9994	0.9994	0.9997
Paclobutrazol	0.437–21.9	0.9994	0.9982	0.9999
Bifenthrin	0.072–3.62	0.9811	0.9998	0.9971
Permethrin	0.607–30.4	0.9915	0.9999	0.9999

**Table 2:** Spiked plant material extractions.

Pesticide	*μ*g/gram plant
Spiked plant material	
Diazinon	6950 ± 5.88
Paclobutrazol	4120 ± 4.46
Bifenthrin	855 ± 3.63
Permethrin	6270 ± 4.69

Data presented as mean *μ*g pesticide/gram plant material ± relative standard deviation. Sample size of 3 for all measurements.

**Table 3:** Recovery of pesticides in smoke condensate.

Sample/residue	*μ*g/gram plant	% Recovery
Water pipe with filters		
Diazinon	589 ± 31.0	0.08
Paclobutrazol	420 ± 32.5	10.2
Bifenthrin	77 ± 34.5	9.00
Permethrin	685 ± 34.9	10.9
Cotton filter		
Diazinon	190 ± 11.0	24.9
Paclobutrazol	109 ± 8.80	30.1
Bifenthrin	20.8 ± 9.16	26.6
Permethrin	134 ± 8.52	25.1
Carbon filter	N/A	N/A
Water pipe w/out filters		
Diazinon	2930 ± 15.1	42.2
Paclobutrazol	2040 ± 11.3	49.5
Bifenthrin	389 ± 10.1	45.4
Permethrin	3760 ± 9.72	59.9
Glass pipe		
Diazinon	4270 ± 12.3	61.5
Paclobutrazol	2789 ± 13.8	67.4
Bifenthrin	516 ± 12.8	60.3
Permethrin	4360 ± 9.70	69.5

Data presented as mean *μ*g pesticide/gram plant material ± relative standard deviation. Sample size of 3 for all measurements.
